# Cytotoxic response against Epstein Barr virus coexists with diffuse large B-cell lymphoma tolerogenic microenvironment: clinical features and survival impact

**DOI:** 10.1038/s41598-017-11052-z

**Published:** 2017-09-07

**Authors:** Melina Cohen, Aldana G. Vistarop, Fuad Huaman, Marina Narbaitz, Fernanda Metrebian, Elena De Matteo, María V. Preciado, Paola A. Chabay

**Affiliations:** 1 Molecular Biology Laboratory, Pathology Division, Ricardo Gutiérrez Children’s Hospital, Buenos Aires, Argentina; 2 Multidisciplinary Institute for Investigation in Pediatric Pathologies (IMIPP), CONICET-GCBA, Buenos Aires, Argentina; 3 Histopathological Laboratory, National Academy of Medicine, Buenos Aires, Argentina; 4 Pathology Division, Ricardo Gutiérrez Children’s Hospital, Buenos Aires, Argentina

## Abstract

Epstein–Barr Virus (EBV) is present in neoplastic cells of 15% of Asian and Latin-American diffuse large B-cell lymphoma (DLBCL) patients. Even though a tolerogenic microenvironment was recently described in DLBCL, little is known concerning immunomodulatory features induced by EBV. As suggested in Hodgkin lymphoma, EBV-specific cytotoxic T-cells are increased but showing immune exhaustion features. Hence, host immunity suppression may play a critical role in tumor progression. This study aimed to investigate, whether an association between tumor microenvironment features and EBV presence is taking place, and its clinical correlate. The incidence of EBV+DLBCL NOS was 12.6% in this cohort. Cytokine and chemokine transcripts expression and immunophenotype analysis showed that EBV infection was associated with increased gene expression of immunosuppressive cytokine (IL-10) together with increased CD8+ T-cells and granzyme B+ cytotoxic effector cells. However, this specific response coexists with a tolerogenic milieu, by PD-1 expression, in EBV+ and EBV−DLBCL cases. High PD-1+ cell counts, EBV presence and low CCL22 expression were associated with worse survival, supporting our hypothesis that EBV-specific response is mounted locally and its inhibition by, for example PD-1+ cells, may negatively affect outcome. The better understanding of the interplay between lymphoma cells and microenvironment in a viral framework could thereby facilitate the discovery of new targets for innovative anti-lymphoma treatment strategies.

## Introduction

In the last few years, the role of EBV in the pathogenesis of diffuse large B cell lymphoma (DLBCL) has become an awkward issue. DLBCL constitutes a very heterogeneous disease with multiple subtypes^1^. Since 2008, World Health Organization (WHO) classification of lymphoid malignancies includes a new provisional entity, EBV-positive DLBCL of the elderly^2^. This DLBCL subtype is defined in people older than 50 years without any known immunodeficiency, characterized by advanced clinical stage and latent EBV infection^3^. EBV-positive DLBCL of the elderly was originally described in Asian populations, with similar prevalence in Latin-American countries as reported subsequently^4–6^. In contrast, lower incidence was found in selected Caucasians populations^7–9^. Clinical studies showed poorer prognosis of patients with EBV-positive DLBCL in elderly patients than their negative counterpart^10, 11^. Of note, various reports demonstrated that EBV-positive DLBCL could also affect younger patients, including pediatric patients, who also showed similar EBV prevalence and comparable histopathological characteristics^6, 12–17^. Furthermore, a poor response to traditional immunochemotherapy was also described^18^. In fact, in the 2016 revision of the WHO classification this new information has led to substitute the modifier “elderly” with “not otherwise specified” (EBV+DLBCL, NOS) in the updated classification, shifting the notion that this entity is limited only to the elderly^19^.

Evading immune response is a recognized hallmark of cancer^20^. Cytotoxic T-cells (CTLs) are a critical component of the immune system. These cells are responsible for killing tumor cells, virus-infected cells and control of persistent and reactivating viral infections. However, persistent antigenic stimulation (e.g., chronic infections), leads to CD8+ T-cell exhaustion, characterized by the induction of a hypoproliferative state and the subsequent loss of the ability to produce antiviral cytokines^21^. As a result, this T-cell exhaustion plays an important role in the development of cancer including hematologic malignancies^22^. For example, PD-1 is expressed by tumor-infiltrating lymphocytes (TILs) in the microenvironment in several hematologic malignancies including follicular lymphoma (FL), DLBCL, and Hodgkin lymphoma (HL)^23–25^.

Diverse mechanisms are employed by EBV-associated lymphomas to suppress T-cell responses^26^. Nevertheless, many reports on several types of lymphomas, with the exception of HL, do not differentiate presence of EBV. This distinction is important since several differences such as the tumor immune microenvironment composition might exist. Less is known about the microenvironment of DLBCL, especially concerning EBV-positive DLBCL. The immune infiltrate in DLBCL comprises innate immune cells like macrophages, dendritic cells, mast-cells, natural killer cells, and adaptive lymphoid cells including T helper cells, along with cytotoxic T- and non-malignant B-cells. In a recent study it was shown that the number of effector/memory T-cells and PD-1-positive cells infiltrating the DLBCL (EBV+ and EBV−) is higher than their counterparts in the peripheral blood, indicating an immune inhibition or escape, despite of EBV presence. Moreover, it was also demonstrated that EBV+ lymphoma cells increase the expression of PD-1 on T-cells, decrease their proliferation and reduce the secretion of several cytokines *in vitro* ^27^. In addition, in a recent report which analyzed a series of DLBCL that included a small subset of DLBCL-NOS, it was observed that EBV-positive cases in young patients evidenced dysregulation of immune checkpoints, indoleamine 2,3-dioxygenase (IDO), and PD-1/PD-L1 axis with promotion of an inhibitory, tolerogenic immune environment^16^.

Growing evidence suggests that host antitumor immunity is suppressed in many ways^28^, and it is assumed to play a critical role in patient outcome. In the present study, we investigated the association between tumor EBV infection, clinical features and the expression of a number of cell markers, cytokines and chemokines within the tumor microenvironment of a cohort of patients with DLBCL from Latin America using molecular techniques and immunohistochemistry methods, to shed light on some aspects of the relationship between infection, tumor and host immunity.

## Results

### Patient characteristics

A total of 102 cases with DLBCL were included in the analysis as the whole cohort, with an age range from 2 to 84 years old (median: 52 years). We also included within this analysis 7 immunocompromised pediatric cases, based on the notion that we have not previously observed any statistical difference in regard of viral expression and preliminary microenvironment composition^17^, also reported by others^29, 30^ (Supplementary Fig. 1). There was no gender predominance, with a male: female ratio of 1:1 approximately (50 males, and 52 females). The diagnosis was achieved in nodal location in 59 cases (58%), while extranodal onset was found in 43 patients (42%). On the basis of Hans IHC classification, 73 cases with enough biopsy material available were classified as GC subtype (n = 34, 47%) and non-GC subtype (n = 39, 53%). The proliferation rate was high, with Ki-67-positive cells representing more than 70% in most cases, reflecting the aggressiveness of this lymphoma. In consequence, 57% of patients presented with advanced stage disease (stage III-IV).

All pediatric patients were treated with the consensus GATLA (Grupo Argentino de Tratamiento de Leucemia Aguda) treatment protocols (9-LNHP-94-GATLA and 1-LNHP-2000 GATLA). Additionally, all adult patients were treated with the consensus according to NCCN, SWOG, MINT, GELA and RICOVER, being the R-CHOP 21 immunotherapy the first line treatment protocol. Full follow-up information on medical records was available for 59 patients (58%), 12 (71%) from the EBV+ group. Patients’ follow-up period ranged from 1 to 157 months (median, 26 months).

### Morphology and Immunophenotype

Morphological heterogeneity was present in almost all specimens of DLBCL, NOS. Mainly, sheets and clusters of monomorphic tumor cells composed of medium to large lymphoid neoplastic cells were observed. These neoplastic B-cells were cytologically diverse and included typically round to oval cells that resemble centroblasts or immunoblasts admixed in some cases with forms resembling Hodgkin/Reed-Sternberg (HRS). Some cases displayed a spectrum in B-cell morphology with higher number of immunoblasts and plasmacytoid differentiation. In a few cases we found a T-cell/Histiocyte-rich Large B-cell lymphoma (THRLBCL)-like pattern, with some mono- or multilobulated tumor cells (HRS-like) and small lymphocytes, plasma cells, eosinophils and histiocytes within the tumor microenvironment. All these cases showed some necrosis foci, half of them with a geographic pattern.

### EBV analysis

EBERs ISH was positive in 17/102 (16.7%) samples found in ≥20% of the viable neoplastic cells. This 20% cut off value was determined based on previous reports^10, 18, 31^ as well as differential viral expression^6^. The median of the % EBERs + cells was 60% in the studied group (range 20–90%). Moreover, these cases displayed latency patterns II and III, as we previously established by IHC for LMP1 and EBNA2 staining (Supplementary Table 2). Immunosuppressed patients are not included in EBV+DLBCL NOS, according to the WHO 2016 classification scheme. Therefore, even though in TILs and cytokines analysis those patients were included within EBV+DLBCL group, specifically to determine EBV+DLBCL incidence, we just considered immunocompetent patients and defined 12/95 (12.6%) EBV+DLBCL NOS in this cohort.

Detail on age, immunological status, viral characteristics and clinical outcome of all EBV+DLBCL cases are described in Supplementary Tables 3 and 4. EBV positivity was more frequently observed in male cases (20% vs. 14%), nodal involvement (10% vs. 12%), advanced clinical stage (20% vs. 5%) and non-GC subtype (23% vs. 21%); however, these differences did not reach statistical significance (p > 0.05, Fisher’s exact test; Supplementary Table 4). The median age of EBV+ cases was 14 years (2-76 years). Furthermore, the mean age of the EBV+ cases was significantly lower than the EBV− patients, (28 vs. 49 years respectively; p = 0.0048, Mann Whitney test). However, when we excluded the immunosuppressed patients as EBV+DLBCL NOS for this comparison, median age of EBV+ cases was 37 years (2-76 years), whereas the mean age of the EBV+ cases and the EBV− patients was not statistically different, (37 vs. 49 years respectively; p = 0.16, Mann Whitney test).

### Cytokines and chemokines gene expression

**Figure 1:**
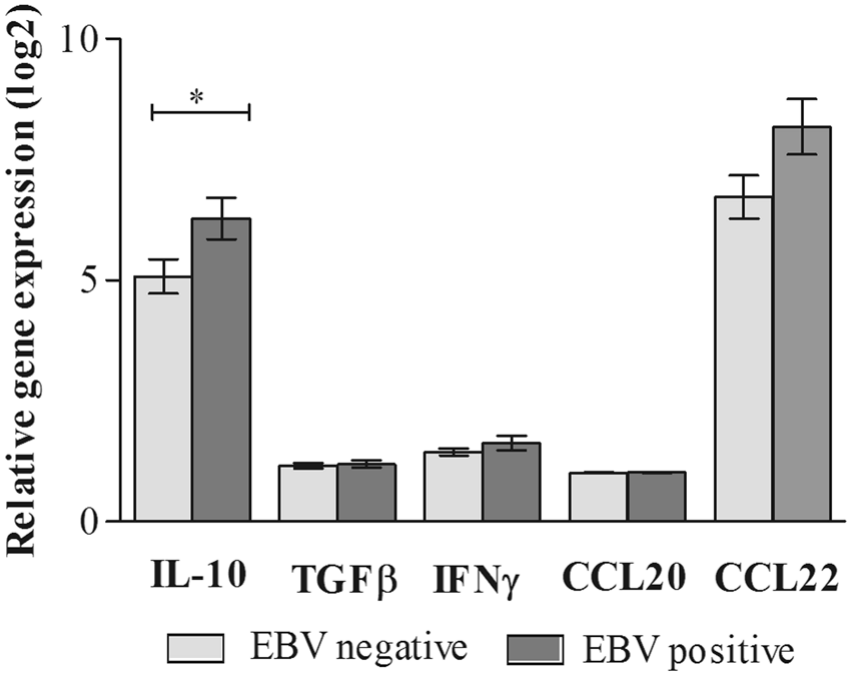
Cytokine and chemokine expression according to EBV status. qPCR was performed to determinate the mRNA relative expression levels of IL-10, TGFβ, IFNγ, CCL20 and CCL22. Endogenous HPRT was used as a reference gene. The bars represent log2 transformed mean ± SE expression levels in DLBCL samples, normalized to mRNA expression in stimulated PBMC cells as calibrator. Light-grey bars indicate EBV−DLBCL cases and dark-grey bars indicate EBV+DLBCL cases. The p value is from Mann-Whitney Test (*p < 0.05).

The transcriptional profile known to be associated with the pro-inflammatory interferon-γ (IFNγ); anti-inflammatory cytokines such as interleukin 10 (IL-10) and tumor growth factor β (TGFβ); and the chemokines (CCL20 and CCL22) were analyzed through RT-qPCR in both EBV+DLBCL and EBV− DLBCL samples (Fig. 1, Supplementary Table 5).

We analyzed how the expression of these genes varied according to EBV status by comparing these two groups, including immunocompromised patients. Interestingly, the results revealed an up-regulation of the log2 transformed mean expression of IL-10 in the EBV-positive tumors. Moreover, we observed a significantly increase in IL-10 expression in EBV+ vs. EBV− cases (mean fold change = 6.3 vs. 5.1 respectively; p = 0.042, Mann Whitney test). On the other hand, despite a trend to an up-regulation of the chemokine CCL22, the mean expression in mRNA levels from the rest of the parameters quantified (TGFβ, IFNγ, CCL20 and CCL22) were not statistically different between groups (p > 0.05, Mann Whitney test).

### Tumor infiltrating lymphocytes (TILs) subsets analysis

**Figure 2:**
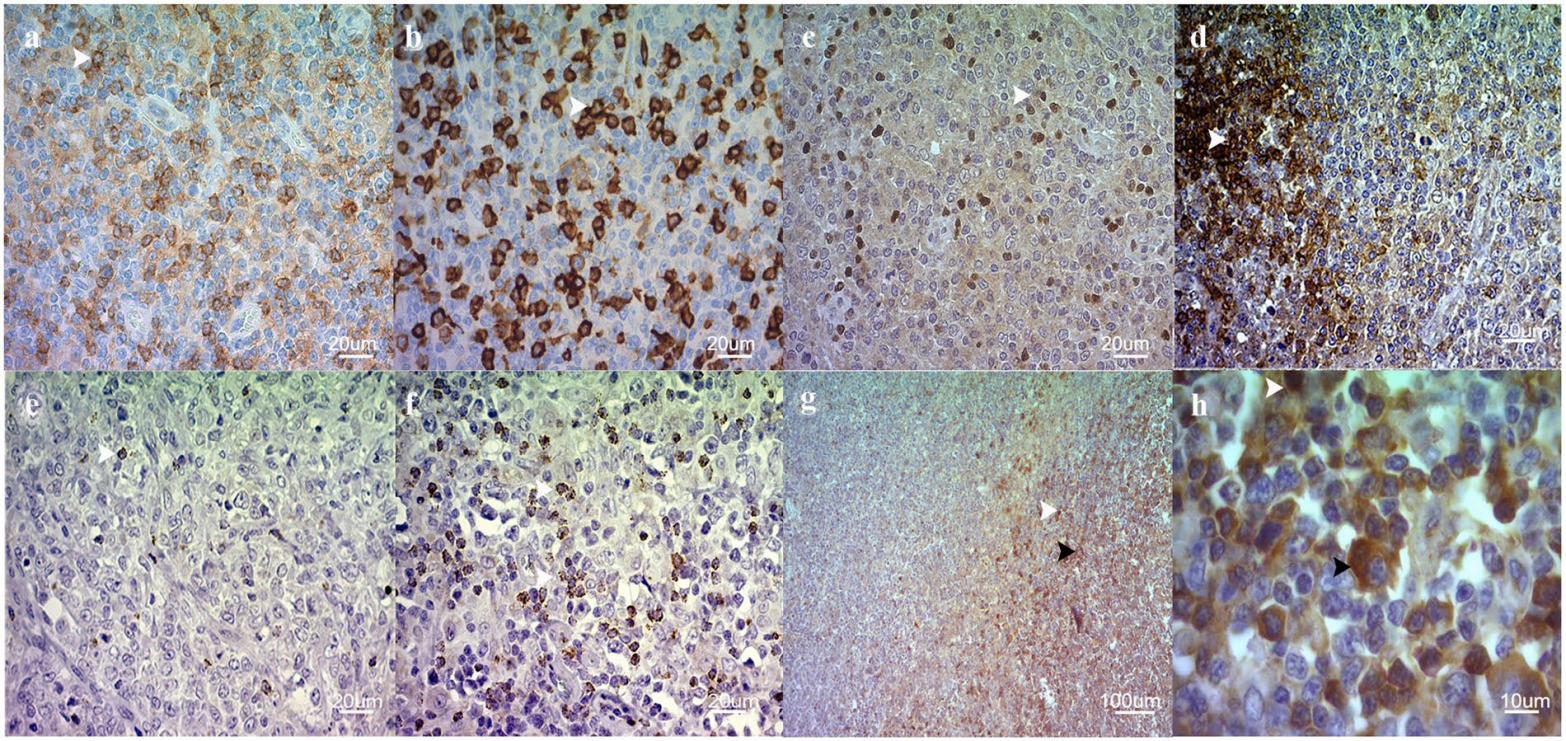
Representative immunohistochemical analysis undertaken on sections from DLBCL biopsies. The expression of membranous CD4 + helper (**a**) and CD8+ cytotoxic (**b**), nuclear Foxp3 + regulatory (**c**), and membranous PD-1+ differentiated (**d**) lymphocytes. GrB+ protein expression in granules of cytotoxic cells displayed a markedly difference between EBV− (**e**) and EBV+DLBCL (**f**). Membranous and often extracellular IL-10 + staining were observed in lymphocytes and neoplastic cells (g and detailed in h). Original magnification: x400 (**a**–**f**), x100 (**g**) and x1000 (**h**). Arrows point out specific staining in representative cells. In panel g and h, white arrows indicate lymphocytes and black arrows indicate neoplastic cells. An immunoperoxidase technique in a paraffin section was used. Digital images were obtained with an Axio CamErc 5 s (Zeiss) camera and acquired using Digital AxioVision Rel. 4.8 image acquisition software.

The representative IHC staining of the different cells markers in the whole DLBCL cohort are exhibited in Fig. 2. The results of the quantitative analysis of subsets of positive TILs denoted a highly heterogeneous expression, both within any given biopsy and among different patient samples. Heterogeneity of expression of all biomarkers is depicted in Supplementary Table 6.

**Figure 3:**
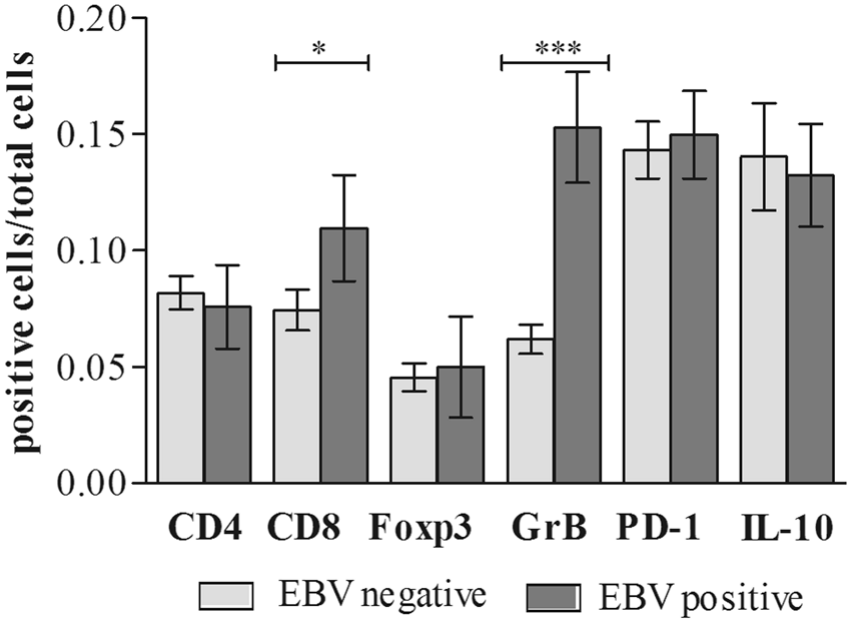
Comparison of TIL subsets (CD4, CD8, Foxp3, GrB, PD-1 and IL-10) analyzed according to EBV status in DLBCL cases. Bar-plot graph showing the mean ± SE distribution of each lymphocyte subset studied in the tumor microenvironment of DLBCL. Light-grey bars indicate EBV− DLBCL cases and dark-grey bars indicate EBV+DLBCL cases. The p value is from Mann-Whitney Test (*p < 0.05, and ***p < 0.001).

In the context of EBV+DLBCL, the virus may modulate the microenvironment by means of cell-mediated immunity. To test this hypothesis, we counted immune cells in EBV+DLBCL biopsies and compared them to EBV− DLBCL. As Fig. 3 shows, the comparison of the 2 groups revealed a difference in cytotoxic cells composition scenario highlighted by CD8+ and GrB+ cells, which were significantly more frequent among EBV+ patients than in the negative counterpart (p = 0.042 and p = 0.0007 respectively, Mann Whitney test), also as exemplified for GrB in Fig. 2e,f. The physiologic regulation of cytotoxic molecules is explained by the fact that GrB is produced in granules upon cytotoxic cell activation^32^. We next sought to determine the contribution of immune tolerogenic microenvironment in DLBCL by means of Foxp3, IL-10 and PD-1 expression, which has been demonstrated to play a role in immune response failure in cHL^33^. PD-1 was expressed in a higher proportion of DLBCL cells as illustrated in Fig. 2d, irrespective of the EBV status (Fig. 3). Additionally, no significant difference was found among the other cellular markers investigated concerning EBV status (Fig. 3, p > 0.05, Mann Whitney test).

### Prognostic impact of EBV status and characteristics of the tumor microenvironment

Clinical outcome was available in 59 patients. The definitions used for this survival analysis are listed in Supplementary information 7. After a median follow-up of 26 months (range 1 to 157 months), 34% of patients died, 56% were alive (30% were alive with stable or progressive disease, and 70% were alive and in complete remission), whereas 10% of patients were lost at follow-up.

**Figure 4:**
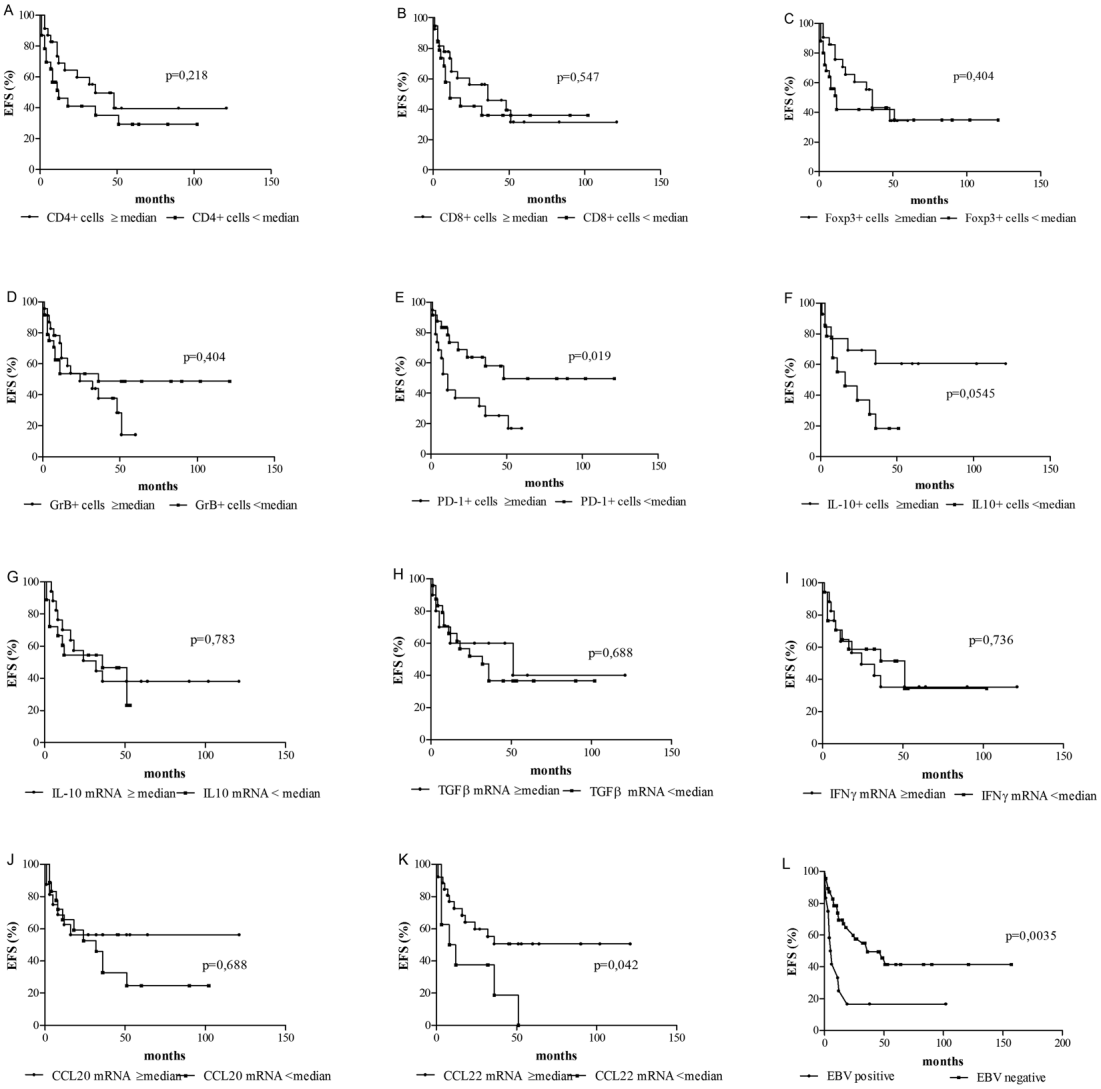
Event-free survival (EFS) analysis of the whole DLBCL cohort related to several tumor microenvironment characteristics and EBV status. The Kaplan-Meier survival curves illustrate the variables associated with the event-free survival in DLBCL. (**A**) CD4 + cells > 0.068 (50^th^ percentile); (**B**) CD8+ cells > 0.054 (50^th^ percentile); (**C**) Foxp3 + cells > 0.024 (50^th^ percentile); (**D**) Granzyme B+ cells > 0.051 (50^th^ percentile); (**E**) PD-1+ cells > 0.131 (50^th^ percentile); (**F**) IL-10 + cells > 0.099 (50^th^ percentile); (**G**) IFNγ > 1.4 (50^th^ percentile); (**H**) IL-10 > 5.5 (50^th^ percentile); (**I**) TGFβ1 > 1.1 (50^th^ percentile); (**J**) CCL20 > 1.0 (50^th^ percentile); (**K**) CCL22 > 6.4 (50^th^ percentile); (**L**) EBV status (positive vs. negative). The p value is from log-rank test (*p < 0.05).

In the whole cohort, 2-year EFS was 51%. Clinical features associated with poor EFS were EBV presence (2-year EFS: 17% vs. 60%, p = 0,0035, log-rank test, Fig. 4L), high PD-1 cells number (>50th percentile; p = 0,0198, log-rank test, Fig. 4E) and low CCL22 transcriptional levels (<50th percentile; p = 0,0415, log-rank test, Fig. 4K). A trend for unfavorable survival was observed in cases with low IL-10 + cell number (<50th percentile; p = 0,0545, log-rank test, Fig. 4F). In contrast, the rest of the variables analyzed were not statistically associated with worse survival (Fig. 4A–D and G–J). Additionally, when the immunosuppressed patients were exclude from the analysis, PD-1 and CCL22 survival analysis remained significant (p = 0,029 and p = 0,0302, respectively; log-rank test) while EBV-associated cases switched to a trend to worse EFS (p = 0,111, log-rank test) (data not shown).

## Discussion

There are only few studies exploring the characteristics of the non-neoplastic milieu in EBV+DLBCL. In this analysis we presented the results of a whole DLBCL cohort from the combination of two reported series, comprising a wide age-range of patients (2–84 years). While using 20% as cutoff value, the prevalence of EBV+DLBCL was 16.7% (17/102), in the whole series, but restricted to 12.6% of patients (12/95), according to WHO 2016 classification scheme. Based on the increasing data regarding EBV presence in young DLBCL patients, the updated classification has led to substitute the modifier “elderly” with “not otherwise specified” (EBV+DLBCL, NOS)^19^. The median age of our EBV+ cases was 14 years when the immunosuppressed patients were included, and raised to 37 years when they were not considered.

The composition of the cytokine and chemokine milieu can have both a positive and a negative influence on the development of adaptive immune responses and modulation of the tumor microenvironment. In addition, several chronic viral infections are associated with the production of increased levels of the immunosuppressive cytokines as IL-10, and also the dysregulation of immune checkpoints, IDO and PD-1/PD-L1 axis with promotion of an inhibitory, tolerogenic immune environment^34^. However, most studies are performed *in vitro* or in peripheral blood lymphocytes that do not exactly reflect the degree of impairment of the immune system at the tumor site; therefore, studies on local cytokine and chemokine composition are required to understand their impact on microenvironment T-cell population homing.

Emerging evidence indicates that EBV has the ability to shape the microenvironment making it more conductive to cell transformation^35^. Our results from cytokine and chemokine gene expression profiling revealed that the most one of the most notable feature in EBV+DLBCL cases was the up-regulation of IL-10 transcripts. Studies performed in EBV+HL showed a similar increment of immunosuppressive cytokine genes (IL-10 and TGFβ) expression. IL-10 is secreted by HRS cells in HL and also by tumor cells and associated macrophages in BL^36^. In our series, we found that IL-10 is produced both by tumor cells and the reactive microenvironment, as shown by IHC, although no statistically significant difference was detected according to EBV status. However, this observation did not correlate with IL-10 transcript expression. It is conceivable that IL-10 IHC staining could be the result of viral and human IL-10 detection, given that commercially available antibodies targeting this cytokine does not differentiate between them, as reported by others^37^, while qPCR analysis is based upon the use of a specific human IL-10 primer pair that does not include sequences homologous to that of the viral IL-10 gene. Thus, our conclusions are based on the endogenous human IL-10 mRNA expression.

It is well known that EBNA1 actively favors the maintenance of the typical HL microenvironment by up-regulating CCL20 to attract Treg to involved tissues^38^. Alternatively, high levels of CCL22, known to strongly attract Treg, were described in all HL cases independently of EBV status^39^. EBV was shown *in vitro* to promote B-cell proliferation by inducing IFNγ expression, mediated by its LMP1 protein^40^. In EBV+DLBCL, this scenario remains quite unexplored. Despite of what we found for IL-10 transcript expression, neither CCL20 nor IFNγ analyzed here showed evidence of any association with EBV presence, in contrast to what was described in HL and *in vitro* studies, respectively. Conversely, CCL22 expression was independent of EBV status, like it was defined for HL cases. Moreover, lack of differences in the T-cell population, especially concerning Treg, analyzed between EBV+ and EBV− DLBCL microenvironment reinforces the abovementioned results. Thus, these observations suggests that, at least in our DLBCL series, the levels of IFNγ, TGFβ, CCL20, CCL22 transcripts could be not necessarily modified by the presence of the virus.

In EBV associated large B-cell lymphomas in young patients, an evidence for a tolerogenic immune environment was revealed by a high prevalence of deregulation of the PD-1/PD-L1 axis^16^. In line with this, in our DLBCL series, we found high PD-1 expression, but irrespective of EBV status. In contrast, as expected, the presence of EBV within the malignant B-cell was associated with homing of CD8+ T lymphocytes, and particularly the cytotoxic GrB+ effector subset. In line with this, a predominantly cytotoxic microenvironment profile characterized by a high number of CD8+ T-cells and GrB expression as was already described in pediatric HL^41^ and a more abundant infiltrate of CD8+ T-cells and NK cells were also detected in adult EBV+HL^42^. On the other hand, a regulatory profile characterized both EBV+ and EBV− HL microenvironment^43^, which coexists with the immune effector cells. Previous reports have suggested that, in patients who develop lymphomas, EBV-specific subsets of circulating T cells are dysfunctional for IFN-γ secretion^44^, showing signs of immune exhaustion in association with increased soluble IL-10 levels^35^. Therefore, in our DLBCL series, cytotoxicity assessed by CD8+ cells and GrB+ expression could not be effective due to immune exhaustion, contributing to the pathogenesis of DLBCL.

In light of the significant heterogeneity of expression of these markers and in order to explore the potential clinical impact of a cytotoxic polarized, activated infiltrate in contrast to a PD-1-expressing suppressive infiltrate, we went on to assess clinical associations. Even though number of cases in this study imposes limitations in relation to the survival analyses, EBV positivity, low CCL22 expression and higher PD-1+ cells were associated with worse survival in the analyzed series. Furthermore, when immunocompromised patients were removed, PD-1 was still a marker of poorer survival, whereas EBV displayed a trend. It was previously demonstrated that the number of PD-1+ TILs correlates with the presence of B symptoms, extranodal sites, and bulky masses in solid tumors^45, 46^, while the presence of a high number of PD-1+ TILs was proved to be a favorable prognostic factor in patients with DLBCL^47^. In contrast, in our series, high PD-1+ cells provide a tolerogenic environment, which may inhibit local tumor specific immune response and ultimately affecting outcome. This exhausted environment in the context of EBV infection might affect local viral specific cytotoxic immune response and probably patient’s survival. This reinforces our hypothesis that in DLBCL patients, an EBV-specific immune response is mounted locally and that inhibition of this immune response by, for example PD-1+ cells, may negatively affect outcome. In addition, reduced homing of immune effector cells induced by lower CCL22 expression might also diminish survival in our series.

The small sample size in the clinicopathological analysis may limit the power to detect differences between groups; hence our findings should be verified in a larger population and validation studies are also required in different geographical locations where age of EBV infection is likely to be later to that seen in Argentina. In addition, it still remains unclear whether expression of PD-1 on tumor cells or on other nonmalignant cells is a key factor associated with clinical outcomes in response to PD-1/PD-L1 blockade in DLBCL. Future functional analysis are necessary to elucidate the complex roles of this pathway. The results correspond to our data, suggesting that PD-1 expression on tumor cells plays a pivotal role in DLBCL tumor microenvironment and contributes to aggressive clinical outcomes, as well as EBV status, and may be an effective therapeutic target.

In summary, this study suggests that tumor microenvironment composition in DLBCL has certain characteristics, given that cytotoxic effector cells are promoted in response to EBV, but this specific immune response coexists with the tolerogenic milieu prevailing in both EBV+ and EBV− DLBCL cases. Additionally, IL-10 expression might contribute to the immunosuppressive environment, even though it is not yet defined whether immune exhaustion is the result of the direct actions of these actors on T-cells. There is likely to be a tumor-mediated T-cell exhaustion mechanism in the DLBCL tumor microenvironment, independently of EBV. Therefore, we hypothesize that regulating the strong local immunosuppression of DLBCL by counteracting PD-1 and its ligands could not only improve the outcomes of EBV+DLBCL immunotherapy, but also improve the efficacy of conventional DLBCL therapy. To the best of our knowledge, this is one of the first reports evaluating this underexplored topic in the case of EBV+DLBCL. There is still much to learn about how the roles of these functional markers (GrB, PD-1 and IL-10) integrate together and with additional parameters that determine the fate of the anti-tumoral/anti-viral response. This is an important point that leads to a better understanding of the interplay between lymphoma cells and their microenvironment in a viral framework. They could thereby facilitate the discovery of new targets for innovative anti-lymphoma treatment strategies. Further *in vitro* and *in vivo* studies will be necessary to evaluate the effects of tolerogenic microenvironment in the context of EBV specific cytotoxic response in DLBCL and their clinical impact.

## Methods

### Patients and samples

For this retrospective study, we included 102 patients combining two previously reported series: 26 pediatric patients belonging to Ricardo Gutiérrez Children’s Hospital and 76 adult patients belonging to National Academy of Medicine, both centers from Buenos Aires, Argentina^6, 17^.

All patients were naïve of treatment at diagnosis. Adult patients had no prior lymphoma or any known underlying immunosuppression. The pediatric patients included 7 immunocompromised (4 had primary immunodeficiency, namely 2 common variable immunodeficiency, 1 Burkley’s syndrome and 1 ataxia-telangiectasia, 2 were PTLD and 1 was HIV+) cases. Formalin-fixed paraffin-embedded (FFPE) consecutive biopsy samples from the corresponding archives of each Histopathological Laboratory were collected, on the basis of the availability of sufficient material, between years 1987–2013 and 2009–2013 for pediatric and adult cases, respectively.

All cases were diagnosed and classified by 4 pathologist (EDM, MN, FM and FH), as DLBCL not otherwise specified (DLBCL, NOS) according to standard WHO criteria^1^. Cases were then subcategorized, when possible, as germinal center (GC) or non-GC according to the algorithm presented by Hans *et al*.^48^. They were evaluated for histopathological features, such as necrosis, mitosis and increase of apoptotic cells.

This study has the approval of the Institutional Review Board and the Ethics Board of National Academy of Medicine and is also in accordance with the Helsinki Declaration of 1975, as revised in 1983. A written informed consent was obtained from all the included patients after the nature of the procedure had been fully explained. The medical records were reviewed with special attention to previous medical history.

### Detection of EBERs by *in situ* hybridization

The Epstein–Barr virus-encoded small RNAs (EBERs) are small non-coding RNAs localized in the nucleus of human cells infected with Epstein–Barr virus (EBV). EBV association in our series of DLBCL was assessed by means of EBERs *in situ* hybridization, which is considered the gold standard method to detect EBV presence in EBV-associated lymphoid malignancies.

An EBERs positive signal is always observed as a dark violet staining (as a result of the degradation of BCIP/NBT substrate by the enzyme alkaline phosphatase) within the nucleus of infected cells, which is exclusively restricted to tumor cells. EBERs *in situ* hybridization (ISH) studies were performed using the PNA *in situ* hybridization detection kit (Dako, Carpinteria, CA) according to the manufacturer’s protocol as previously described, and cases were considered as EBV positive using a cutoff of 20% of positive nuclear staining restricted to tumor cells^6^.

### Immunophenotyping and cell counting

Immunohistochemistry (IHC) staining for B-cell lymphoma differential diagnosis and tumor microenvironment immunophenotyping was performed on FFPE tissue sections with a panel of antibodies: CD3 (Cell Marque, Rocklin, CA), CD20 (Dako), CD10 (Cell Marque, AR, USA), bcl-2 (Dako), bcl-6 (Dako), MUM1 (Dako), Ki67 (Dako), CD4 for T helper (Th) (Leica, Newcastle, UK), CD8 for CTL (Dako), Foxp3for T regulatory (Treg) (abcam, Cambridge, UK) as previously described^6^ with the addition of granzyme B (GrB)for activated cytotoxic cells (clone GB7, AbD Serotec, Oxford, UK), PD-1 (CD279) for exhausted T-cells (AbD Serotec) and IL-10 (Abcam) for anti-inflammatory cytokine productive cell markers. The counting of microenvironment CD4, CD8, Foxp3, GrB, PD-1 and IL-10 positive cells was performed as follows: numbers of total or immunopositive cells per high-power field (HPF) were counted by two independent observers blinded to the characteristics of each subject, in 10 fields selected on the basis of the best-preserved tissue areas. Enumeration was performed directly on the computer screen. To avoid double counting or omission of positive cell when a large number were present, the image was computer saved and counting was performed using the point selection facility of the Image J software (NIH, Bethesda, MD, USA). The expression of the results was defined as number of immunopositive cells divided by the total number of counted cells. Cells partly included in the fields were not counted. For quality control, normal tonsillar lymphoid tissue was used as positive controls. Negative controls for each case consisted of substituting the primary antibody with buffer.

### Cell isolation and stimulation

Heparinized peripheral blood (10 ml) from healthy donors was collected and pooled. Peripheral blood mononuclear cells (PBMC) were separated from whole blood by Ficoll-Paque plus (GE Healthcare, Sweden) density gradient centrifugation. Cells were washed twice with phosphate-buffered saline (PBS) and isolated PBMC were cultured in RPMI 1640 medium supplemented with 10%heat-inactivated fetal bovine serum (FBS; Natocor, Argentina), 50ug/ml Gentamicin Sulfate and GlutaMAX™ supplement(“RPMI complete medium”, all supplements from Invitrogen, California, USA), at 37 °C in 5%CO2.

Cells were stimulated by placing 2 × 10^6^ lymphocytes/ml in culture medium with PMA (Sigma-Aldrich, St Louis, MO, USA, 100 ng/ml) and ionomycin (Sigma-Aldrich, 1 μg/ml). They were incubated for 5 hours at 37 °C. After harvest, cells were washed as above and stored at -70 °C prior to RNA isolation as calibrators as well as positive controls for gene expression studies.

### RNA extraction and qPCR

Approximately 2 × 10 μm sections from each of the FFPE biopsy samples were used for nucleic acid extraction and purification. Total RNA was purified using the RecoverAll Total Nucleic Acid Isolation Kit (Ambion, Texas, USA) and 2 μg of total RNA was reverse transcribed using Superscript II RT kit (Invitrogen) according to the manufacturer’s instructions. To remove contaminated DNA in total RNA, a DNAse step was included for all RNA samples. RNA from stimulated PBMC used as calibrators was extracted by MasterPure™ RNA Purification Kit (Epicentre, Madison, WI, USA) according to the manufacturer’s instructions. The specific primers for target genes: cytokines (IL-10, TGFβ1 and IFNγ) and chemokines (CCL20 and CCL22); and the endogenous HPRT gene were obtained from http://primerdepot.nci.nih.gov/. qPCR was performed and validated as previously described^17^ using a StepOne real-time detection system (Applied Biosystems, Foster City, CA). Specific human IL-10 primer pair does not include sequences homologous to that of the viral IL-10 gene. The normalized transcription values were calculated by the Pfaffl Method^49^. The data of the gene expression were further log2 transformed. The mean value of the Ct of an equivalent quantity of RNA input from stimulated PBMC was used as calibrator.

### Statistical analysis

Statistical analysis was performed using GraphPad Prism 5 (GraphPad Software Inc., San Diego California USA). Categorical variables were analyzed using Fisher’s exact test or Chi Square test when necessary. Mann–Whitney test was used to compare the means between groups in relation to EBV presence. All tests were two-tailed, and p < 0.05 was considered statistically significant.

The follow-up time was defined as the length of time from the date of diagnosis to either last follow-up date or when a given event occurs. Event-free survival (EFS) was measured from the date of diagnosis and first treatment to either date of disease progression or discontinuation of treatment for any reason or censoring date when patient’s loss of contact or withdrawal from the study. More extensive definitions of common terms in survival analysis are listed in Supplementary information 7. For survival analyses related to reactive microenvironment expression, two groups were considered: the group of patients with expression values above the 50^th^ percentile (median) versus the group below this point. This cut-point was arbitrarily chosen for cell counts as well as for transcripts expression, and the resulting absolute cut-off values were above or equal to 0.068, 0.054, 0.024, 0.051, 0.131 and 0.099 for CD4 + , CD8+, Foxp3 + , GrB+, PD-1+ and IL-10 + cells, respectively; and 1.4, 5.5, 1.1, 1.0 and 6.4 for IFNγ, IL-10, TGFβ1, CCL20 and CCL22 mRNA levels, respectively. Kaplan-Meier curves based on the abovementioned median thresholds were generated and statistical significance of each marker was determined using the log-rank test. Two-sided P values less than 5% were considered statistically significant and P values less than 10% were considered of borderline significance. Given the small number of cases, multivariate Cox analysis to determine the independent prognostic value of statistically significant variables in univariate analyses was not performed.

### Data Availability

All data generated or analyzed during this study are included in this published article (and its Supplementary Information files).

## Electronic supplementary material

Supplementary Information
