# Supplementary material for: Cytotoxic response against Epstein Barr virus coexists with diffuse large B-cell lymphoma tolerogenic microenvironment: clinical features and survival impact
Source: Sci Rep. 2017 Sep 7;7:10813. doi: 10.1038/s41598-017-11052-z (PMC5589929; doi:10.1038/s41598-017-11052-z)
Supplement: Supplementary file 1 — Supplementary Information [file 41598_2017_11052_MOESM1_ESM.pdf]

**Title:** Cytotoxic response against Epstein Barr virus coexists with diffuse large B-cell lymphoma tolerogenic microenvironment: clinical features and survival impact

**Authors:** *Melina Cohen[1,2], Aldana G. Vistarop[1,2], Fuad Huaman[3], Marina Narbaitz[3], Fernanda Metrebian[3], Elena De Matteo[4], María V. Preciado[1,2] and Paola A. Chabay[1,2]

**Affiliations:**[1]Molecular Biology Laboratory, Pathology Division, Ricardo Gutiérrez Children's Hospital, Buenos Aires, Argentina; [2] Multidisciplinary Institute for Investigation in Pediatric Pathologies (IMIPP), CONICET-GCBA, Buenos Aires, Argentina; [3]Histopathological Laboratory, National Academy of Medicine,Buenos Aires, Argentina; and [4] Pathology Division, Ricardo Gutiérrez Children's Hospital, Buenos Aires, Argentina.

***Corresponding author:** Melina Cohen, PhD.

Laboratorio de Biología Molecular, División de Patología, Hospital de Niños R. Gutiérrez. Instituto Multidisciplinario de Investigación en Patologías Pediátricas (IMIPP), CONICET-GCBA.

Gallo 1330. C1425EFD. Buenos Aires, Argentina

Pho/Fax: 5411-49629138.

E-mail: melucohen@yahoo.com.ar

**Supplementary Figure 1. Comparative analysis of immunological markers regarding age.**

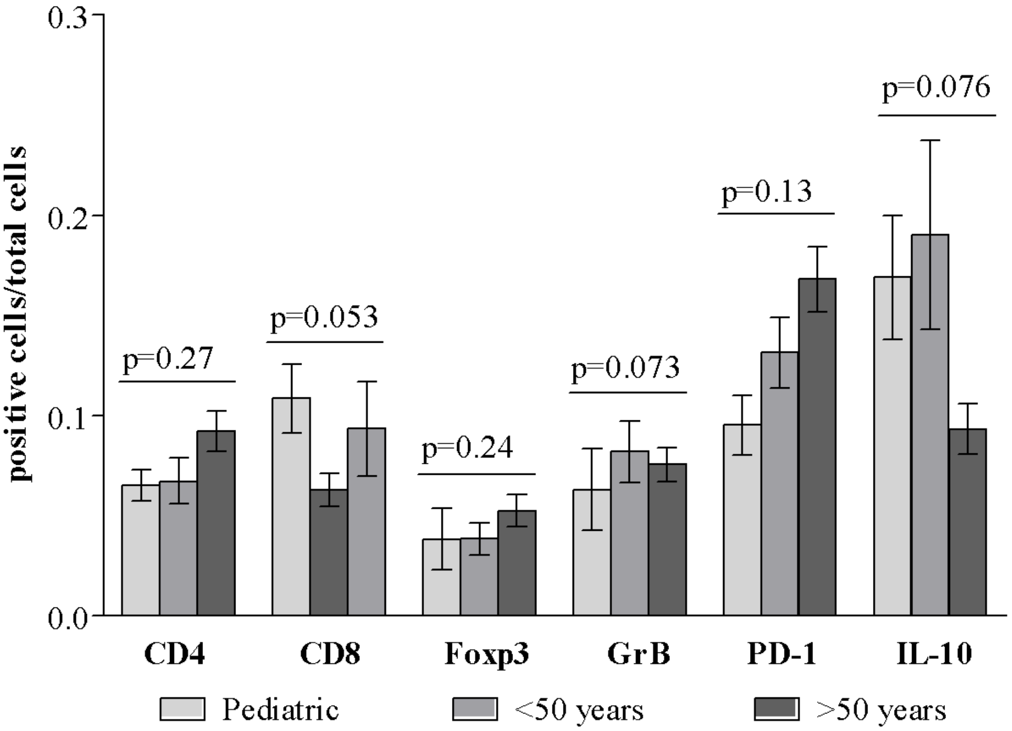


Since immunosenescence has been postulated to be implicated in the pathogenesis, we investigated if there was any difference in tumor microenvironment composition associated with age. All the immunological markers studied were discriminated according to age (pediatric, older and younger than 50 years old patients), and we did not find statistical differences among them (CD4 p=0.27, CD8 p=0.053, Foxp3 p=0.24, GrB p=0.0703, PD1 p=0.13, IL10 p=0.075; Kruskal-Wallis test). Therefore, based on those results, we decided to not discriminate our series into different age groups for cell markers analysis.

**Supplementary Table 2. EBERs ISH detection and types of latency pattern expression on EBV+ DLBCL cases**

| | EBERs[1] | Latency I | Latency II | Latency III | Latency II-III |
|---|---|---|---|---|---|
| **no. positive cases/total (%)** | 17/102 (17) | 3/17 (18) | 7/17 (41) | 3/17(18) | 4/17 (24) |

1EBV status determined by EBERs ISH (≥20% EBERs+ cells as a cutoff value). Latency pattern defined by EBERs ISH, LMP1 and EBNA2 IHC results. Latency II-III was defined when could not be discriminate due to lack of material.

**Supplementary Table 3. Types of latency pattern on EBV+ DLBCL cases related to clinical outcome**

| Patient nº | Age (years) | Immunological status | ISH EBERs[1] | % | Latency expression[2] | Outcome |
|---|---|---|---|---|---|---|
| 1 | 2 | IC | + | 40 | III | Dead |
| 2 | 9 | IS | + | 70 | III | Dead |
| 3 | 3 | IC | + | 30 | III | Alive |
| 4 | 4 | IS | + | 60 | II/III | Dead |
| 5 | 3 | IS | + | 80 | II/III | Dead |
| 6 | 14 | IS | + | 70 | II/III | Dead |
| 7 | 7 | IS | + | 30 | III | Dead |
| 8 | 8 | IC | + | 90 | I | Alive |
| 9 | 2 | IC | + | 50 | III | LF |
| 10 | 18 | IC | + | 90 | III | Dead |
| 11 | 38 | IC | + | 90 | II | LF |
| 12 | 35 | IC | + | 80 | II/III | Dead |
| 13 | 44 | IC | + | 50 | II | LF |
| 14 | 68 | IC | + | 80 | II | Dead |
| 15 | 76 | IC | + | 30 | II | Dead |
| 16 | 76 | IC | + | 25 | II | LF |
| 17 | 73 | IC | + | 20 | III | LF |

Abbreviations: IS, immunosuppressed. IC, immunocompetent. ISH, in situ hybridization. LF, lost at follow-up. 1≥20% EBERs+ tumor cells as a cut-off value. 2 indicates latency pattern defined by IHC.

**Supplementary Table 4. Demographic and histological characteristics of DLBCL series related to EBV status (positive vs. negative)**

| Patients' characteristics | EBV+[1] n (%) | EBV- n (%) | p[2] |
|---|---|---|---|
| **Age (years)** | | | |
| pediatric | 4 (21) | 15 (79) | |
| < 50 | 4 (17) | 20 (83) | 0.26 |
| ≥ 50 | 4(8) | 48(92) | |
| **Gender** | | | |
| male | 10 (20) | 40 (80) | 0,43 |
| female | 7 (14) | 45 (86) | |
| **Histologicalsubtype** | | | |
| GC | 7 (21) | 27(79) | 0,075 |
| post-GC | 9 (23) | 30 (77) | |
| ND | 1 | 28 | |
| **Clinicalstage** | | | |
| I-II | 1 (5) | 21 (95) | 0,23 |
| III-IV | 5 (20) | 20 (80) | |
| ND | 11 | 44 | |
| **Primarysite** | | | |
| nodal | 11 (21) | 42 (79) | 0,30 |
| extranodal | 6 (12) | 43 (88) | |

1EBV status determined by EBERs ISH (≥20% EBERs+ cells as a cutoff value).2p as determined by Fisher's or Chi square exact test. Abbreviations: GC: germinal-centre; ND: not determined (insufficient material).

**Supplementary Table 5. Cytokines and chemokines gene expression related to EBV expression status**

| Gene | EBV status† | Mean‡ | SE | Median‡ | Range | *p** |
|---|---|---|---|---|---|---|
| **IL-10** | Negative | 5.1 | 0.35 | 5.2 | (1.0-8.8) | 0.042* |
| | Positive | 6.3 | 0.43 | 6.2 | (3.9-9.2) | |
| **TGFβ** | Negative | 1.2 | 0.056 | 1.1 | (1.0-2.9) | 0.80 |
| | Positive | 1.2 | 0.076 | 1.0 | (1.0-2.0) | |
| **IFNγ** | Negative | 1.4 | 0.066 | 1.4 | (1.0-2.5) | 0.18 |
| | Positive | 1.6 | 0.15 | 1.5 | (1.0-3.0) | |
| **CCL20** | Negative | 1.0 | 0.0025 | 1.0 | (1.0-1.1) | 0.69 |
| | Positive | 1.0 | 0.0084 | 1.0 | (1.0-1.1) | |
| **CCL22** | Negative | 6.7 | 0.44 | 7.4 | (1.0-10) | 0.11 |
| | Positive | 8.2 | 0.58 | 8.3 | (4.7-11) | |

SE: standard error †EBV status determined by EBERs ISH (≥20% EBERs+ cells as a cutoff value). ‡The number represents the mean log2 transformed of the gene expression data. *p as determined by Mann Whitney

**Supplementary Table 6. TIL subsets quantification in tumor microenvironment related to EBV status**

| TIL subsets | EBV status† | Mean‡ | SE | Median | Range | *p** |
|---|---|---|---|---|---|---|
| **CD4** | Negative | 0.082 | 0.007 | 0.069 | (0-0.29) | 0.77 |
| | Positive | 0.076 | 0.02 | 0.062 | (0-0.22) | |
| **CD8** | Negative | 0.074 | 0.009 | 0.049 | (0-0.51) | 0.042* |
| | Positive | 0.11 | 0.02 | 0.12 | (0-0.24) | |
| **Foxp3** | Negative | 0.045 | 0.006 | 0.024 | (0-0.23) | 0.61 |
| | Positive | 0.050 | 0.02 | 0.018 | (0-0.29) | |
| **GrB** | Negative | 0.062 | 0.006 | 0.041 | (0-0.26) | 0.0007* |
| | Positive | 0.15 | 0.02 | 0.17 | (0.02-0.28) | |
| **PD-1** | Negative | 0.14 | 0.01 | 0.12 | (0-0.53) | 0.38 |
| | Positive | 0.15 | 0.02 | 0.13 | (0.07-0.29) | |
| **IL-10** | Negative | 0.128 | 0.02 | 0.0984 | (0.02-0.39) | 0.41 |
| | Positive | 0.162 | 0.03 | 0.162 | (0.05-0.32) | |

SE: standard error †EBV status determined by EBERs ISH (≥20% EBERs+ cells as a cutoff value). ‡The number represents the mean of the n°+ cells / nº total cells.*p as determined by Mann Whitney test.

## Supplementary information 7. Definitions of common terms in survival analysis

Event: Death, disease occurrence, disease recurrence, recovery, or other experience of interest. In this particular study an Event is defined as non-response, death from any cause, tumor progression or second malignancy.

Time: The time from the beginning of an observation period (such as surgery or beginning treatment) to (i) an event, or (ii) end of the study, or (iii) loss of contact or withdrawal from the study.

Censoring / Censored observation: If a subject does not have an event during the observation time, they are described as censored. The subject is censored in the sense that nothing is observed or known about that subject after the time of censoring. A censored subject may or may not have an event after the end of observation time.

Event-free survival: According to the National Cancer Institute (NCI), after primary treatment for a cancer ends, EFS is the measurement of the length of time that the patient remains free of certain complications or events that the treatment was intended to prevent or delay. These events may include the return of the cancer or the onset of certain symptoms, such as bone pain from cancer that has spread to the bone. In a clinical trial, measuring the event-free survival is one way to see how well a new treatment works.

Follow-up: the date when the patient go to the medical consultation for any cause.

Time to event: the quantity of time (generally in months) from the diagnosis to the observation defined previously as event (non-response, death from any cause, tumor progression or second malignancy).
